# Crystal structure of *catena*-poly[*N*,*N*,*N*′,*N*′-tetra­methyl­guanidinium [(chlorido­cadmate)-di-μ-chlorido]]

**DOI:** 10.1107/S2056989015020836

**Published:** 2016-01-01

**Authors:** Mamadou Ndiaye, Abdoulaye Samb, Libasse Diop, Thierry Maris

**Affiliations:** a Laboratoire des Produits Naturels, Département de Chimie, Faculté des Sciences et Techniques, Université Cheikh Anta Diop, Dakar, Senegal; b Laboratoire de Chimie Minérale et Analytique, Département de Chimie, Faculté des Sciences et Techniques, Université Cheikh Anta Diop, Dakar, Senegal; c Département de Chimie, Université de Montréal, 2900 Boulevard Édouard-Montpetit, Montréal, Québec, H3C 3J7, Canada

**Keywords:** crystal structure, five-coordinated cadmium, N—H⋯Cl hydrogen bonds, polyanionic chain

## Abstract

In the structure of the title salt, {(C_5_H_14_N_3_)[CdCl_3_]}_*n*_, the Cd^II^ atom of the complex anion is five-coordinated by one terminal and four bridging Cl atoms. The corresponding coordination polyhedron is a distorted trigonal bipyramid, with Cd—Cl distances in the range 2.4829 (4)–2.6402 (4) Å. The bipyramids are condensed into a polyanionic zigzag chain extending parallel to [101]. The tetra­methyl­guanidinium cations are situated between the polyanionic chains and are linked to them through N—H⋯Cl hydrogen bonds, forming a layered network parallel to (010).

## Abstract 2

The structure of the title salt, {(C_5_H_14_N_3_)[CdCl_3_]}_*n*_, consists of infinite zigzag polyanionic ^1^_∞_[CdCl_4/2_Cl_1/1_]^−^ chains held together by tetra­methyl­guanidinium cations through N—H⋯Cl hydrogen bonds, leading to a layered structure parallel to (010).

## Chemical context   

Tetra­methyl­guanidine is known to crystallize in its neutral form, as a Lewis base or as a singly protonated cation. Several cationic complexes of Pd, Ga and Pt have been reported with tetra­methyl­guanidine acting as a ligand (Li *et al.*, 2005 ▸; Cowley *et al.*, 2005 ▸; Eliseev *et al.*, 2013 ▸), and halogenidometalates have been reported with tetra­methyl­guanidinium as a counter-cation (Bujak *et al.*, 1999 ▸; Bujak & Zaleski, 2007 ▸). Since none of these complexes has cadmium as a component, we decided to study the inter­actions between tetra­methyl­guanidine and [CdCl_2_]·H_2_O, which has yielded the title salt, (C_5_H_14_N_3_)^+^[CdCl_3_]^−^, (I).
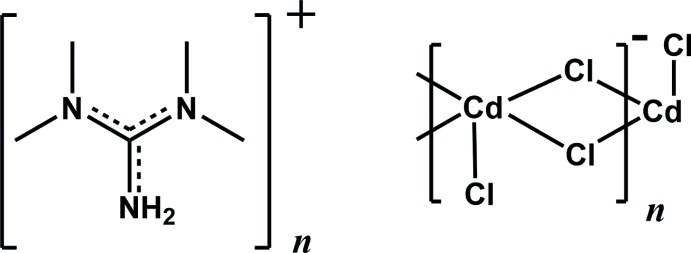


## Structural commentary   

**Figure 1:**
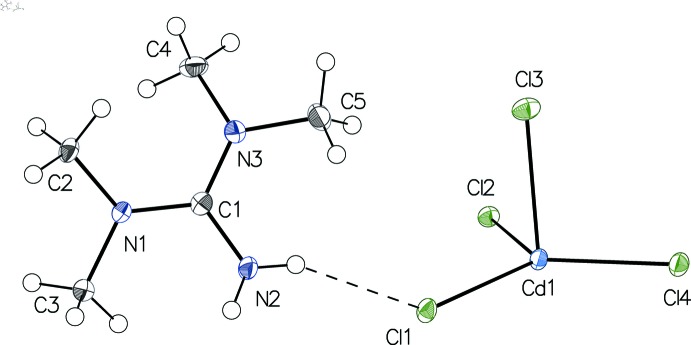
The asymmetric unit of compound (I), with displacement ellipsoids drawn at the 50% probability level. The N—H⋯Cl hydrogen bond is indicated by a dashed line.

The asymmetric unit of (I) (Fig. 1 ▸) consists of a Cd^II^ cation surrounded by four Cl atoms and one *N,N,N′,N′*-tetra­methyl­guanidinium cation. The coordination polyhedron around Cd^II^ can be described best as a distorted trigonal bipyramid where atoms Cl1, Cl2 and Cl4 define the equatorial plane while atoms Cl3 and Cl4^i^ [symmetry code: (i) 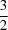 − *x*, 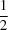 − *y*, 1 − *z*] are in axial positions with a Cl3—Cd1—Cl4^i^ angle of 166.347 (10)°. The equatorial Cd—Cl bond lengths range from 2.4829 (4) Å to 2.5829 (4) Å while the axial bond lengths Cd1—Cl3 and Cd1—Cl4^i^ are 2.5854 (4) Å and 2.6403 (4) Å, respectively. The CdCl_4_ moieties of the asymmetric unit are related by an inversion center, generating an extended zigzag chain of edge-sharing trigonal bipyramids running parallel to [101]. These ^1^_∞_[CdCl_4/2_Cl_1/1_]^−^ chains are formed by the bridging atoms Cl2, Cl3, Cl4 and Cl4^i^ with a Cd—Cd—Cd angle of 137.893 (6)°. The corrugation of the chains results in rather short Cd⋯Cd distances of 3.8720 (3) and 3.8026 (3) Å. The same kind of zigzag chain is found, for example, in the [CdCl_3_]^−^ salt obtained with benzyl­tri­ethyl­ammonium as counter-cation (Sun & Jin, 2013 ▸) but with a less pronounced corrugation. Accordingly, the angle between two successive rectangular [Cd_2_Cl_2_] units is 57.928 (3)° in the structure of the benzyl­tri­ethyl­ammonium compound compared with 129.859 (2)° for the present structure. The tetra­methyl­guanidinium cation has the central atom C1 in an almost trigonal–planar configuration. The three N—C—N angles range from 119.26 (14) to 121.14 (14)° and the r.m.s deviation from the least-squares plane calculated with atoms C1, N1, N2 and N3 is only 0.005 Å. The corresponding C—N bond lengths of 1.330 (2), 1.3360 (19), and 1.3441 (19) Å indicate a partial double-bond character. Hence the positive charge may be considered as delocalized in the CN_3_ plane (Tiritiris, 2012 ▸). The two pairs of di­methyl­ammonium groups are twisted by 24.67 (8) and 27.31 (9)° with respect to this plane.

## Supra­molecular features   

**Figure 2:**
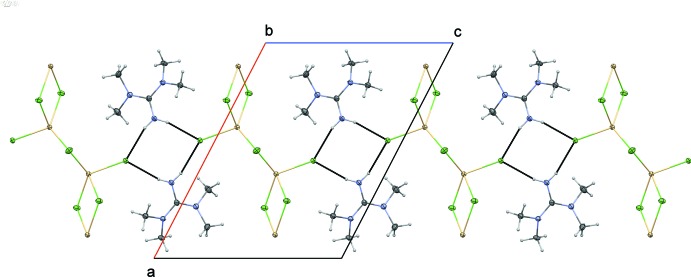
Partial packing diagram of (I), viewed along [010], showing one layer made up of alternating ^1^_∞_[CdCl_4/2_Cl_1/1_]^−^ chains and inter­mediate tetra­methyl­guanidinium cations. N—H⋯Cl hydrogen bonds are shown as black dotted lines.

The ^1^_∞_[CdCl_4/2_Cl_1/1_]^−^ chains are inter­connected through N—H⋯·Cl hydrogen bonds by pairs of tetra­methyl­guanidinium cations linked to symmetry-related Cl1 atoms (Table 1 ▸). These inter­actions define layers extending parallel to (010) (Fig. 2 ▸).

## Database survey   

The tri­chlorido­cadmate anion, [CdCl_3_]^−^, may have various discrete or chain structures with tetra­hedral, octa­hedral and trigonal–bipyramidal environments around the central Cd^II^ cation. A search in the Cambridge Structural Database (CSD Version 5.36 with three updates; Groom & Allen, 2014 ▸) returned only five entries with the chains having a trigonal–bipyramidal environment for Cd^II^. The corresponding structures contain different cations such as sulfonium ylide (Sabounchei *et al.*, 2013 ▸), tetra­ethyl­ammonium (Lakshmi *et al.*, 2004 ▸), hexa­decyl sulfonium (Sokka *et al.*, 2008 ▸), benzyl­tri­ethyl­ammonium (Sun & Jin, 2013 ▸) or tri­methyl­ammonium­phenyl-4-thiol (Tang & Lang, 2011 ▸).

## Synthesis and crystallization   

Crystals suitable for a single-crystal X-ray diffraction study were obtained by mixing stoichiometric amounts of tetra­methyl­guanidine with CdCl_2_·H_2_O in ethanol and allowing the solvent to evaporate slowly at room temperature.

## Refinement   

Crystal data, data collection and structure refinement details are summarized in Table 2 ▸. The H-atom positions of all methyl groups were placed geometrically and refined with *U*_iso_(H) = 1.5*U*_eq_(C). H atoms bonded to the N atoms were located from a Fourier difference map and were refined freely.

## Supplementary Material

Crystal structure: contains datablock(s) I. DOI: 10.1107/S2056989015020836/wm5207sup1.cif

Structure factors: contains datablock(s) I. DOI: 10.1107/S2056989015020836/wm5207Isup2.hkl

CCDC reference: 1434977

Additional supporting information:  crystallographic information; 3D view; checkCIF report

## Figures and Tables

**Table 1:** Hydrogen-bond geometry (Å, °)

*D*—H⋯*A*	*D*—H	H⋯*A*	*D*⋯*A*	*D*—H⋯*A*
N2—H2*A*⋯Cl1	0.83 (2)	2.51 (2)	3.2871 (15)	157 (2)
N2—H2*B*⋯Cl1^i^	0.83 (2)	2.46 (2)	3.2710 (15)	164 (2)

Symmetry code: (i) 
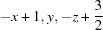
.

**Table 2:** Experimental details

Crystal data	
Chemical formula	(C_5_H_14_N_3_)[CdCl_3_]
*M*_r_	334.94
Crystal system, space group	Monoclinic, *C*2/*c*
Temperature (K)	100
*a*, *b*, *c* (Å)	15.1305 (7), 14.2921 (6), 11.6939 (5)
β (°)	117.370 (2)
*V* (Å^3^)	2245.69 (17)
*Z*	8
Radiation type	Ga *K*α, λ = 1.34139 Å
μ (mm^−1^)	14.50
Crystal size (mm)	0.19 × 0.10 × 0.10

Data collection	
Diffractometer	Bruker Venture Metaljet
Absorption correction	Multi-scan (*SADABS*; Krause *et al.*, 2015 ▸)
*T*_min_, *T*_max_	0.400, 0.752
No. of measured, independent and observed [*I* > 2σ(*I*)] reflections	24837, 2593, 2575
*R*_int_	0.043
(sin θ/λ)_max_ (Å^−1^)	0.650

Refinement	
*R*[*F* ^2^ > 2σ(*F* ^2^)], *wR*(*F* ^2^), *S*	0.016, 0.040, 1.13
No. of reflections	2593
No. of parameters	122
H-atom treatment	H atoms treated by a mixture of independent and constrained refinement
Δρ_max_, Δρ_min_ (e Å^−3^)	0.63, −0.40

Computer programs: *APEX2* and *SAINT* (Bruker, 2014 ▸), *SHELXT* (Sheldrick, 2015*a* ▸), *SHELXL2014* (Sheldrick, 2015*b* ▸), *OLEX2* (Dolomanov *et al.*, 2009 ▸), *Mercury* (Macrae *et al.*, 2008 ▸) and *publCIF* (Westrip, 2010 ▸).

## supplementary crystallographic information

### Crystal data

**Table:**

(C_5_H_14_N_3_)[CdCl_3_]	*F*(000) = 1312
*M**_r_* = 334.94	*D*_x_ = 1.981 Mg m^−^^3^
Monoclinic, *C*2/*c*	Ga *K*α radiation, λ = 1.34139 Å
*a* = 15.1305 (7) Å	Cell parameters from 9941 reflections
*b* = 14.2921 (6) Å	θ = 3.9–60.7°
*c* = 11.6939 (5) Å	µ = 14.50 mm^−^^1^
β = 117.370 (2)°	*T* = 100 K
*V* = 2245.69 (17) Å^3^	Block, clear light colourless
*Z* = 8	0.19 × 0.10 × 0.10 mm

### Data collection

**Table:**

Bruker Venture Metaljet diffractometer	2593 independent reflections
Radiation source: Metal Jet, Gallium Liquid Metal Jet Source	2575 reflections with *I* > 2σ(*I*)
Helios MX Mirror Optics monochromator	*R*_int_ = 0.043
Detector resolution: 10.24 pixels mm^-1^	θ_max_ = 60.7°, θ_min_ = 3.9°
ω and φ scans	*h* = −19→19
Absorption correction: multi-scan (*SADABS*; Krause *et al.*, 2015)	*k* = −18→18
*T*_min_ = 0.400, *T*_max_ = 0.752	*l* = −15→15
24837 measured reflections	

### Refinement

**Table:**

Refinement on *F*^2^	Primary atom site location: structure-invariant direct methods
Least-squares matrix: full	Hydrogen site location: mixed
*R*[*F*^2^ > 2σ(*F*^2^)] = 0.016	H atoms treated by a mixture of independent and constrained refinement
*wR*(*F*^2^) = 0.040	*w* = 1/[σ^2^(*F*_o_^2^) + (0.0144*P*)^2^ + 2.6083*P*] where *P* = (*F*_o_^2^ + 2*F*_c_^2^)/3
*S* = 1.13	(Δ/σ)_max_ = 0.001
2593 reflections	Δρ_max_ = 0.63 e Å^−^^3^
122 parameters	Δρ_min_ = −0.40 e Å^−^^3^
0 restraints	

### Special details

**Table:**

Experimental. X-ray crystallographic data for I were collected from a single-crystal sample, which was mounted on a loop fiber. Data were collected using a Bruker Venture diffractometer equipped with a Photon 100 CMOS Detector, a Helios MX optics and a Kappa goniometer. The crystal-to-detector distance was 4.0 cm, and the data collection was carried out in 1024 *x* 1024 pixel mode.
Geometry. All e.s.d.'s (except the e.s.d. in the dihedral angle between two l.s. planes) are estimated using the full covariance matrix. The cell e.s.d.'s are taken into account individually in the estimation of e.s.d.'s in distances, angles and torsion angles; correlations between e.s.d.'s in cell parameters are only used when they are defined by crystal symmetry. An approximate (isotropic) treatment of cell e.s.d.'s is used for estimating e.s.d.'s involving l.s. planes.

### Fractional atomic coordinates and isotropic or equivalent isotropic displacement parameters (Å^2^)

**Table:**

	*x*	*y*	*z*	*U*_iso_*/*U*_eq_	
N1	0.18403 (9)	0.36639 (9)	0.56127 (12)	0.0134 (2)	
N2	0.35116 (10)	0.36153 (11)	0.61584 (14)	0.0191 (3)	
N3	0.24068 (10)	0.42244 (9)	0.41914 (13)	0.0150 (3)	
C1	0.25851 (11)	0.38434 (10)	0.53204 (15)	0.0132 (3)	
C2	0.09488 (11)	0.42502 (11)	0.51613 (15)	0.0153 (3)	
H2C	0.1042	0.4816	0.4756	0.023*	
H2D	0.0835	0.4425	0.5894	0.023*	
H2E	0.0373	0.3901	0.4532	0.023*	
C3	0.19647 (12)	0.29906 (11)	0.66197 (15)	0.0152 (3)	
H3A	0.2479	0.2535	0.6719	0.023*	
H3B	0.1334	0.2664	0.6379	0.023*	
H3C	0.2164	0.3322	0.7435	0.023*	
C4	0.14557 (12)	0.41074 (12)	0.30272 (15)	0.0200 (3)	
H4A	0.1074	0.3604	0.3163	0.030*	
H4B	0.1581	0.3948	0.2300	0.030*	
H4C	0.1076	0.4692	0.2842	0.030*	
C5	0.32011 (13)	0.46786 (12)	0.40087 (17)	0.0212 (3)	
H5A	0.3718	0.4902	0.4841	0.032*	
H5B	0.2926	0.5210	0.3420	0.032*	
H5C	0.3491	0.4228	0.3643	0.032*	
Cd1	0.60861 (2)	0.27596 (2)	0.41882 (2)	0.01199 (5)	
Cl1	0.54707 (3)	0.30385 (3)	0.57950 (3)	0.01619 (8)	
Cl2	0.5000	0.15363 (3)	0.2500	0.01344 (10)	
Cl3	0.5000	0.39855 (4)	0.2500	0.01852 (11)	
Cl4	0.76617 (3)	0.35865 (2)	0.45006 (4)	0.01607 (8)	
H2A	0.3944 (15)	0.3603 (16)	0.591 (2)	0.021 (5)*	
H2B	0.3677 (16)	0.3540 (16)	0.694 (2)	0.026 (6)*	

### Atomic displacement parameters (Å^2^)

**Table:**

	*U*^11^	*U*^22^	*U*^33^	*U*^12^	*U*^13^	*U*^23^
N1	0.0108 (6)	0.0151 (6)	0.0146 (6)	0.0020 (5)	0.0061 (5)	0.0016 (5)
N2	0.0120 (6)	0.0333 (8)	0.0139 (7)	0.0037 (5)	0.0076 (5)	0.0038 (6)
N3	0.0148 (6)	0.0161 (6)	0.0152 (6)	0.0008 (5)	0.0079 (5)	0.0010 (5)
C1	0.0143 (7)	0.0120 (7)	0.0141 (7)	0.0012 (5)	0.0072 (6)	−0.0027 (5)
C2	0.0118 (7)	0.0156 (7)	0.0196 (7)	0.0026 (5)	0.0083 (6)	−0.0001 (6)
C3	0.0140 (7)	0.0174 (7)	0.0151 (7)	0.0016 (6)	0.0073 (6)	0.0020 (6)
C4	0.0200 (8)	0.0249 (8)	0.0124 (7)	0.0067 (6)	0.0052 (6)	0.0027 (6)
C5	0.0232 (8)	0.0210 (8)	0.0254 (8)	0.0017 (6)	0.0163 (7)	0.0050 (7)
Cd1	0.00895 (7)	0.01692 (7)	0.01027 (7)	−0.00065 (3)	0.00457 (5)	−0.00147 (3)
Cl1	0.01164 (16)	0.02608 (19)	0.01180 (16)	0.00064 (14)	0.00620 (13)	−0.00316 (14)
Cl2	0.0121 (2)	0.0156 (2)	0.0115 (2)	0.000	0.00446 (18)	0.000
Cl3	0.0168 (2)	0.0176 (2)	0.0151 (2)	0.000	0.00209 (19)	0.000
Cl4	0.01132 (16)	0.01586 (16)	0.02072 (18)	−0.00045 (12)	0.00710 (14)	0.00232 (13)

### Geometric parameters (Å, º)

**Table:**

N1—C1	1.3441 (19)	C4—H4A	0.9800
N1—C2	1.4649 (19)	C4—H4B	0.9800
N1—C3	1.4643 (19)	C4—H4C	0.9800
N2—C1	1.330 (2)	C5—H5A	0.9800
N2—H2A	0.83 (2)	C5—H5B	0.9800
N2—H2B	0.83 (2)	C5—H5C	0.9800
N3—C1	1.3360 (19)	Cd1—Cl1	2.4829 (4)
N3—C4	1.467 (2)	Cd1—Cl2	2.5829 (4)
N3—C5	1.465 (2)	Cd1—Cl3	2.5854 (4)
C2—H2C	0.9800	Cd1—Cl4	2.5323 (4)
C2—H2D	0.9800	Cd1—Cl4^i^	2.6403 (4)
C2—H2E	0.9800	Cl2—Cd1^ii^	2.5830 (4)
C3—H3A	0.9800	Cl3—Cd1^ii^	2.5854 (4)
C3—H3B	0.9800	Cl4—Cd1^i^	2.6402 (4)
C3—H3C	0.9800		
			
C1—N1—C2	122.68 (13)	N3—C4—H4B	109.5
C1—N1—C3	121.14 (12)	N3—C4—H4C	109.5
C3—N1—C2	114.98 (12)	H4A—C4—H4B	109.5
C1—N2—H2A	118.6 (14)	H4A—C4—H4C	109.5
C1—N2—H2B	121.6 (15)	H4B—C4—H4C	109.5
H2A—N2—H2B	120 (2)	N3—C5—H5A	109.5
C1—N3—C4	122.47 (13)	N3—C5—H5B	109.5
C1—N3—C5	121.21 (14)	N3—C5—H5C	109.5
C5—N3—C4	115.83 (13)	H5A—C5—H5B	109.5
N2—C1—N1	119.26 (14)	H5A—C5—H5C	109.5
N2—C1—N3	119.57 (14)	H5B—C5—H5C	109.5
N3—C1—N1	121.14 (14)	Cl1—Cd1—Cl2	111.033 (10)
N1—C2—H2C	109.5	Cl1—Cd1—Cl3	98.115 (10)
N1—C2—H2D	109.5	Cl1—Cd1—Cl4^i^	95.516 (13)
N1—C2—H2E	109.5	Cl1—Cd1—Cl4	118.137 (13)
H2C—C2—H2D	109.5	Cl2—Cd1—Cl3	85.262 (13)
H2C—C2—H2E	109.5	Cl2—Cd1—Cl4^i^	89.152 (12)
H2D—C2—H2E	109.5	Cl3—Cd1—Cl4^i^	166.347 (10)
N1—C3—H3A	109.5	Cl4—Cd1—Cl2	130.701 (9)
N1—C3—H3B	109.5	Cl4—Cd1—Cl3	91.126 (11)
N1—C3—H3C	109.5	Cl4—Cd1—Cl4^i^	83.088 (12)
H3A—C3—H3B	109.5	Cd1—Cl2—Cd1^ii^	94.798 (17)
H3A—C3—H3C	109.5	Cd1^ii^—Cl3—Cd1	94.679 (18)
H3B—C3—H3C	109.5	Cd1—Cl4—Cd1^i^	96.910 (13)
N3—C4—H4A	109.5		
			
C2—N1—C1—N2	148.47 (15)	C4—N3—C1—N1	−27.1 (2)
C2—N1—C1—N3	−33.2 (2)	C4—N3—C1—N2	151.23 (15)
C3—N1—C1—N2	−18.4 (2)	C5—N3—C1—N1	161.27 (14)
C3—N1—C1—N3	159.92 (14)	C5—N3—C1—N2	−20.4 (2)

Symmetry codes: (i) −*x*+3/2, −*y*+1/2, −*z*+1; (ii) −*x*+1, *y*, −*z*+1/2.

### Hydrogen-bond geometry (Å, º)

**Table:**

*D*—H···*A*	*D*—H	H···*A*	*D*···*A*	*D*—H···*A*
C2—H2*C*···Cl4^iii^	0.98	2.87	3.6567 (16)	138
C3—H3*B*···Cl1^iv^	0.98	2.92	3.7614 (16)	144
C4—H4*B*···Cl4^ii^	0.98	2.87	3.8347 (17)	169
N2—H2*A*···Cl1	0.83 (2)	2.51 (2)	3.2871 (15)	157 (2)
N2—H2*B*···Cl1^v^	0.83 (2)	2.46 (2)	3.2710 (15)	164 (2)

Symmetry codes: (ii) −*x*+1, *y*, −*z*+1/2; (iii) −*x*+1, −*y*+1, −*z*+1; (iv) −*x*+1/2, −*y*+1/2, −*z*+1; (v) −*x*+1, *y*, −*z*+3/2.
